# p-Synephrine: an overview of physicochemical properties, toxicity, biological and pharmacological activity

**DOI:** 10.17179/excli2024-8088

**Published:** 2025-03-05

**Authors:** Maciej Kulawik, Kaja Bajewska, Anna Kulawik

**Affiliations:** 1 Poznan University of Medical Sciences, Fredry 10 St., 61-701 Poznan, Poland; 2 Doctoral School, Poznan University of Medical Sciences, Bukowska 70 St., 60-812 Poznan, Poland; 3 Department of Pharmacognosy and Biomaterials, Faculty of Pharmacy, Poznan University of Medical Sciences, 3 Rokietnicka St., 60-806 Poznan, Poland

**Keywords:** p-synephrine, phytochemicals, Citrus species, dietary supplements

## Abstract

p-Synephrine is a popular ingredient in dietary supplements. It is also found in trace amounts in living organisms. It is advertised as a weight loss supplement and it is supposed to improve performance in sports. It is contained in plants of the Citrus family, making it widespread in the human diet. Its pharmacological properties include effects on multiple receptors and signaling pathways. Its effects on the alpha and betanergic systems promote doubts about its safety. There are many studies describing a lack of concern when it comes to the potential harmful effects of this compound. On the other hand, several health incidents associated with p-synephrine use have been reported in the scientific literature, making the toxicity of this compound unclear. This review aims to organize the current knowledge about p-synephrine, including physicochemical characteristics, sources of occurrence, pharmacological effects and possible toxic effects. In addition, the presence of three substitution isomers of the hydroxyl group and one chiral carbon atom causes confusion in the literature. Studies conducted on the short-term use of p-synephrine do not indicate its toxicity at low doses for healthy people. Further studies are needed to determine its long-term safety and possible interactions with other chemical compounds.

See also the graphical abstract(Fig. 1).

## Synephrine

**Figure 2:**
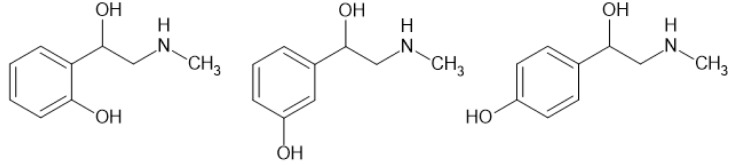
Chemical structures of o-, m- and p-synephrine

Synephrine is a group of three isomeric substances differing in the position of the hydroxyl group connected with the aromatic ring in phenylethanolamine structure (Rossato et al., 2010[77]; Stohs et al., 2011[89]). Depending on the place of substituent, there are three molecules: orto-, meta-, and para-synephrine (see Figure 2(Fig. 2)). Due to the presence of a chiral carbon atom to which a hydroxyl group is attached in the aliphatic chain, it is possible to have two enantiomers: d- and l- for each of the 3 described compounds. By such a large number of possible isomers, 6 compounds are included in the synephrine group (Allison et al., 2005[1]; Rossato et al., 2010[77]). Another name for this compound is sympathol (respectively p-sympathol, etc.) (Bergmann and Sulzbacher, 1951[7]). m-l-Synephrine is also known as a phenylephrine (Stohs and Preuss, 2012[87]). Phenylephrine has been confused in the literature with p-synephrine. Therefore, it is worthwhile to ensure which isomer was used (Bouchard et al., 2004[10]; Nasir et al., 2004[63]). The d- isomer is dextrorotatory and corresponds to the (S) configuration, while the l- isomer is levorotary and has the (R) configuration (Midgley et al., 1989[60]). Freebase l-p-synephrine melts at 162-164 °C with decomposition, the most accurate measurements by differential scanning calorimetry show a melting point of racemic p-synephrine at 199.8 °C (Stewart et al., 1964[85]; Rosa et al., 2016[74]). p-Synephrine hydrochloride melts at 150-152 °C (Bergmann and Sulzbacher, 1951[7]). p-d-Synephrine crystalizes in the orthorhombic crystal system (Ianno et al., 2020[39]).

When exposed to UV light, p-synephrine can degrade. The main product of this process is 1-methyl-2,3-dihydro-1H-indole-3,6-diol. The formation of this product is attributed to the activity of the amine group. The resulting product in a study on human lymphocytes was characterized by genotoxicity and reduced cell viability (Malesuik et al., 2024[58]).

p-Synephrine and bitter orange extract are ingredients in dietary supplements. They are advertised as weight loss remedies (Fugh-Berman and Myers, 2004[25]; Rossato et al., 2011[76]). p-Synephrine use has been banned by sports organizations, including: The National Collegiate Athletic Association, Major League Baseball and The National Football League (Piattoly, 2022[69]).

## Sources of p-Synephrine

Plants are the most common source of p-synephrine. In addition, it occurs in animal tissues and cell lines.

p-Synephrine is the phytochemical in the *Citrus *species (*Rutaceae*)*.* It is found in fruits and flowers of some citruses such as Bitter orange (*Citrus aurantium*), Pomelo (*Citrus grandis*), Cleopatra mandarin (*Citrus reshni*), Japanese mandarin (*Citrus unshiu*) and others (Wheaton and Stewart, 1969[101]; Arbo et al., 2008[2]; Dragull et al., 2008[24]; Lin et al., 2023[53]). It is assumed that only *para* enantiomer occurs in citruses (Stohs, 2013[86]). However, the older scientific literature is uncertain about the natural occurrence of only one compound (Allison et al., 2005[1]). The most commonly reported compound in the literature is para-l-synephrine, which is found in plants.

**Figure 3:**
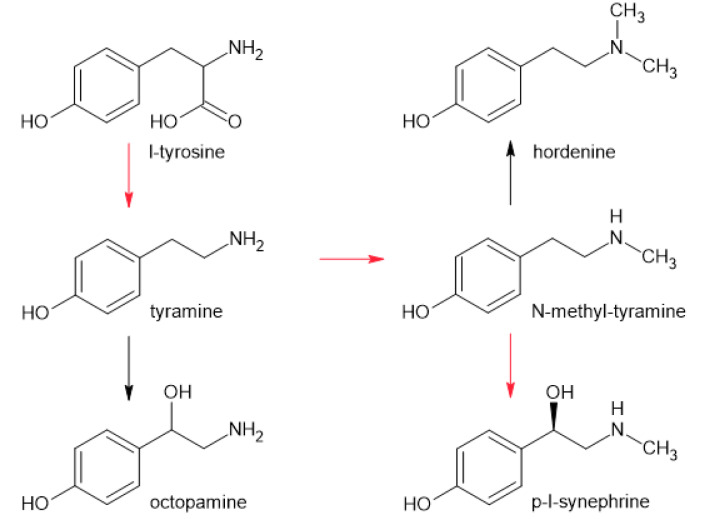
Biosynthesis of p-l-synephrine. The first step is decarboxylation, the second is methylation, and the last is hydroxylation. Tyramine could be hydroxylated to octopamine. N-methyl-tyramine could also be methylated to hordenine.

The biosynthesis reaction substrate is l-tyrosine (Bartley et al., 2010[6]). However, small amounts of the d-isomer can be formed during extraction or processing fruits (Stohs and Preuss, 2012[87]). Synephrine racemization is affected by factors such as pH and temperature. Therefore, extraction should be carried out under controlled conditions to prevent racemization. Previous literature reports of finding p-d-synephrine in plants may be due to poorly performed analyses or isomerization during fruit processing (Pellati et al., 2010[67]). To prevent erroneous analytical results, enantioselective methods are available for the identification of synephrine isomers in biological samples (Pellati and Benvenuti, 2007[66]). Wheaton and Stewart (1969[101]) studied the biosynthesis of synephrine in *Citrus reshni*. Radio-traced studies with tyramine as a substrate showed a major pathway of phenolic amine synthesis (see Figure 3(Fig. 3)) (Wheaton and Stewart, 1969[101]). Key genes that may be responsible for biosynthesis have been identified (Zhong et al., 2024[109]).

A naturally biosynthesized compound is p-l-synephrine. Synthetic p-synephrine is a racemic mixture of l- and d-enantiomers (Stohs and Preuss, 2012[87]). With this information, it is possible to determine whether the synephrine found in dietary supplements comes from plant sources (Koh et al., 2021[47]). The molecule could also be used as a urinary biomarker for citruses consumption (Bader et al., 2017[4]). It is also a potential biomarker of the authenticity of orange honey (Tette et al., 2017[93]). p-Synephrine was identified in the urine collected from horses. Its source was the *Eragrostis* and *Lucerne* hay that the animals consumed (Brewer et al., 2022[12]). p-Synephrine is sometimes called an elusive or trace amine. That is because the described molecule was found in the human body in plasma and platelet pellets in low quantities (D'Andrea et al., 2003[18][19]; Arbo et al., 2008[2]). p-Synephrine was detected in trace amounts in the urine of people who did not consume citruses in the last 12 hours, which may indicate the endogenous source of the discussed substance (Watson et al., 1990[100]). Research on patients with migraines showed that the level of trace amines (including p-synephrine) was higher than in the control group (D'Andrea et al., 2006[17]). Trace amines may act as neurotransmitters but p-synephrine's physiological role in the brain has not been understood (Arbo et al., 2008[2]).

**Figure 4:**
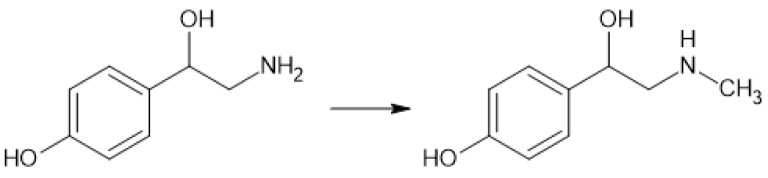
Possible pathway for p-synephrine biosynthesis in the human body from p-octopamine by PNMT

Levels of p-synephrine in Wistar rats were measured while studying the effects of dolutegravir on the trace amine profile (Henning et al., 2024[33]). Dolutegravir is an antiviral drug and an HIV integrase inhibitor. It is also proposed as an anti-tumor drug (Hou et al., 2024[37]). Synephrine was found in the urine of dolutegravir-treated rats as well as in the control group. In the dolutegravir-treated group, the concentration of synephrine in urine was slightly lower but still statistically significant (Henning et al., 2024[33]). p- and m-Synephrine were also detected in the Caco-2 immortalized cell line (human colorectal adenocarcinoma) and cardiomyocytes collected from an adult rat (Rossato et al., 2010[77]). Determinable amounts of p-synephrine indicate its endogenous source. Unfortunately, there have been no studies on its biosynthesis in the human body. It is believed to be formed by methylation of p-octopamine by phenylethanolamine N-methyltransferase (PNMT) (see Figure 4(Fig. 4)) (Lindemann and Hoener, 2005[54]).

## Pharmacological Activity

Synephrine is a similar molecule to endogenous amines like epinephrine or norepinephrine. p-Synephrine interacts mainly with alpha (α) and beta (β) adrenergic receptors. Localization of the hydroxyl group affects the binding strength of the substance to the α and β receptors (Stohs et al., 2011[89]). It also shows an affinity for 5-HT_1D_, 5-HT_2A_ and 5-HT_6_ receptors, trace amine-associated receptor 1 (TAAR1) and Neuromedin U2 receptor (NMU2R) (Wainscott et al., 2007[97]; Hibino et al., 2009[35]; Zheng et al., 2014[108]; Koh et al., 2019[46]; Wang et al., 2025[98]). However, attention should be paid to the impermeability of the blood-brain barrier to p-synephrine. This property was verified using the SwissADME online tool (http://www.swissadme.ch/index.php, accessed on 23 January 2025) (Daina et al., 2017[20]). The poor permeability of the test compound to the brain could limit the concentration of the described compound in the cerebrospinal fluid. This will result in marginal effects on receptors located in the brain. It should be remembered that in some diseases the blood-brain barrier can increase its permeability, resulting in greater penetration of certain substances into the brain (Dong, 2018[23]). However, a study on mice showed that p-synephrine can cross the blood-brain barrier (Wang et al., 2025[98]). This contradicts the *in silico* simulation. Further studies are needed to see if the described compound can cross this barrier in humans. Table 1(Tab. 1) (References in Table 1: Berry et al., 2017[8]; Hering et al., 2020[34]; Hibino et al., 2009[35]; Hoyer, 2019[38]; Karila et al., 2015[43]; Koh et al., 2019[46]; Ma et al., 2010[56], 2014[57]; Perez 2021[68]; Rutigliano et al., 2018[78]; Samanta et al., 2024[79]; Saunders and Limbird, 1999[80]; Tanveer et al., 2024[92]; Wachter and Gilbert, 2012[96]; Wainscott et al., 2007[97]; Wang et al., 2025[98]; Yuan et al., 2024[106]; Zhang and Stackman, 2015[107]; Zheng et al., 2014[108]) summarizes the effects of synephrine on individual receptors. Adrenergic receptors are distributed throughout the human body. Among other things, they are responsible for stress response, regulation of heart rate, blood pressure and metabolic rate. They are stimulated by catecholamines such as adrenaline and norepinephrine. There are 9 receptors of these types in humans. Receptors of the adrenergic system can be divided into 3 groups: α1, α2 and β. Each group is divided into 3 subtypes (Wu et al., 2021[103]; Xanthopoulos et al., 2021[104]; Kraboth and Kalman, 2023[49]). p-Synephrine acts on α_1A_, α_2B_, α_2C_ and β1, β2, β3 receptors (Carpéné et al., 1999; Stohs et al., 2011[89]; Yuan et al., 2024[106]).

### In vitro studies

Research on the human embryonic kidney (HEK293) cell line showed that p-synephrine is a partial agonist of the α_1A_ adrenergic receptor. Maximal response to the receptor was given at a concentration of 100 µM. A study on the Chinese ovary hamster (CHO) cell line showed an antagonistic effect on α_2B_ and α_2C_ receptors (Ma et al., 2010[55]).

The risk of inducing arrhythmias by p-synephrine was studied on human induced pluripotent stem cell-derived cardiomyocytes (hiPSC-CMs) and freshly isolated rat cardiomyocytes. The study showed that the risk of inducing arrhythmias by p-synephrine through ingestion of doses used in dietary supplements is low. The risk of arrhythmia increases at concentrations higher than 200 µM. An attempt to reverse changes in cardiomyocyte contractility was carried out using blockers selectively targeting β1, β2 and β3 receptors. β1 blockers almost completely reversed the changes induced by p-synephrine, indicating the affinity of the test compound mainly toward these receptors. In addition, the study showed that p-synephrine is more reactive towards human cardiomyocytes than rat cardiomyocytes (Yuan et al., 2024[106]).

p-Synephrine is a highly potent agonist of NMU2R (Zheng et al., 2014[108]). Central administration of neuromedin U is known to inhibit appetite by affecting NMU2R. Other molecules that interact with NMU2R can also regulate appetite (Bhattacharyya et al., 2004[9]). This may explain the use of p-synephrine during weight loss (Zheng et al., 2014[108]).

p-Synephrine is an antagonist of TAAR1, but its affinity is very low (Wainscott et al., 2007[97]). Research on the effects of p-synephrine on TAAR1 is scarce, but the latest results indicate that p-synephrine may affect other TAAR receptor subtypes (Koh et al., 2019[46]). In humans TAAR1 plays a role in regulation of the reward system, cognitive processes and mood regulation (Nair et al., 2022[62]).

A study on mitochondria in the rat brain showed that synephrine can be metabolized by monoamine oxidase (MAO) types A and B. MAO type A showed higher activity (Suzuki et al., 1979[90]).

p-Synephrine is transported by OCT1 and OCT3. In rat cardiomyocytes, it did not reduce GSH levels compared to m-synephrine, which generated more ROS. p-Synephrine penetrated cells in greater amounts than m-synephrine (Rossato et al., 2011[75]).

A study on rat skeletal myoblasts (L6) showed no cytotoxicity of p-synephrine after 24 hours of incubation. A concentration of p-synephrine in the medium higher than 25 µM was shown to increase glucose consumption by cells. This is followed by increased lactic acid production. The study also showed that the tested compound increased the amount of the glucose transporter Glut4 after 2 hours incubation (Hong et al., 2012[36]). Another study on rat hepatoma cells (H4IIE) tested how p-synephrine affects glucose profusion by cells. Adding p-synephrine to glucose-free medium at concentrations of 1; 5; 25; 100 µM reduces glucose production by cells 24 hours after administration. Incubation of cells for 3 days in a p-synephrine-containing medium showed no cytotoxicity. It was tested whether inhibition of alpha and beta receptors could affect the aforementioned effects of the tested substance. The results indicate that p-synephrine suppresses glucose production by a different mechanism than interaction with the adrenergic system. This study also found that p-synephrine reduces glucose production by affecting serine/threonine kinase other than kinases A, C and G. In addition to this, the test compound was found to have little effect on the lipid profile of cells (Cui et al., 2015[16]).

A study on RAW267.7 macrophage mouse cell line showed the anti-inflammatory activity of p-synephrine. Cells were stimulated with lipopolysaccharide (LPS). p-Synephrine inhibits p38 MAPK and NF-κB pathways. Research on LPS-induced systemic inflammatory response syndrome (SIRS) mouse model revealed that p-synephrine given orally decreased proinflammatory cytokine levels in the serum. It was also connected with a higher survival rate of SIBS mice (Ishida et al., 2022[40]). Another study on NF-κB and MAPK pathways was prepared on a mouse model of diabetes induced by alloxan injection. Administration of p-synephrine reduced the level of free radicals, increased glutathione level in the serum, improved glucose tolerance and reduced tissue insulin resistance. p-Synephrine also decreased inflammation in renal tissue; this activity could be caused by the regulation gene expression of tumor necrosis factor-α (TNF-α), interleukin 6 and interleukin 1β. The anti-inflammatory effects are likely connected with the regulation of MAPK and NF-κB pathways caused by synephrine (Wang et al., 2023[99]). An *in vivo* study in a mouse model of acute lung injury induced by LPS administration, confirmed the anti-inflammatory properties of p-synephrine (5 and 10 mg/kg body weight, intraperitoneally before LPS administration). In this study, p-synephrine decreased TNF-α and IL-6, and increased IL-10. In the p-synephrine-treated group, lower amounts of the reactive oxygen species (ROS) in the lung tissue were measured, tissue edema and histopathological changes were also less significant. In addition, p-synephrine inhibited NF-κB phosphorylation (Wu et al., 2014[102]).

p-Synephrine shows anti-adipogenic activity on 3T3-L1 cell line. This effect is due to regulation of the Akt pathway and suppression of adipogenesis-related proteins (Guo et al., 2019[26]).

Research on the effects of p-synephrine on cancer is lacking. It has been shown that p-synephrine inhibits the proliferation, migration, colony formation and invasion capacity of esophageal squamous cell carcinoma (ESCC). Synephrine can downregulate galactin-3 expression by inactivating the AKT and ERK pathways. The sensitivity of cancer cells to 5-fluorouracil was also increased. An *in vivo* study in nude mice confirmed the anti-tumor effect of p-synephrine on ESCC tumor xenografts at doses of 20 mg/kg of animal body weight (Xu et al., 2018[105]). p-Synephrine can bind to DNA through hydrophobic interactions. In lung cancer cells (H460), p-synephrine reduced their viability due to increased expression of Bax and p53 proteins. A decrease in PI3K, AKT and mTOR mRNA was also observed. This indicates a reduction in cancer cell proliferation through the interaction of the compound with the PI3K/AKT/ mTOR pathway (Taheri et al., 2022[91]).

The largest study on how p-synephrine alters gene expression was published in 2020. Tests were conducted on a Caco-2 cell line and human stomach mucosa cells (MNP01). p-Synephrine at concentrations of 2-200 μM had no cytotoxic or mutagenic effects on both cell lines. The 24-hour incubation increased the amount of ROS in both cell lines, while it also increased the amount of GSH. This correlation may explain the lack of cytotoxicity. In addition, p-synephrine alters gene expression in the cells tested. In MNP01 cells, it upregulated the expression of ADCY3, MAPK1, JUN and AKT1, while it downregulated the expression of TNF gene. In the Caco-2 cell line, it upregulated the expression of MAPK1, GNAS, JUN, RELA, AKT1, PRKACA, PRKAR2A genes. Genes such as JUN and AKT1 are associated with proliferation, while RELA and TNF are associated with inflammation (Ribeiro et al., 2021[72]).

A study on the 3T3 cell line showed that administration of p-synephrine inhibited interleukin-4 (IL-4) induced eotaxin-1 production. In contrast, it did not inhibit TNF-α-induced eotaxin-1 secretion. This indicates a specific inhibition of eotaxin-1 secretion caused by IL-4. The inhibition of eotaxin-1 secretion was caused by the inhibition of STAT6 phosphorylation by p-synephrine in the JAK/STAT signaling pathway (Roh et al., 2014[73]).

### Studies in humans

A study of tritium-labeled p-synephrine showed its pharmacokinetics and metabolism in humans. After oral administration, the highest plasma concentration was reached after 1-2 hours. The biological half-life was about 2 hours. With intravenous and oral administration, about 80 % of the radiation was recorded in urine. Two thirds of the tritium in urine was in p-hydroxymandelic acid (Hengstmann and Aulepp, 1978[32]).

The study on 13 athletes showed no change in sports performance such as a squat jump, a countermovement jump, a 15-second repeated jump test, followed by 60-meter and 100-meter sprint contests. The subjects were given 3mg of p-synephrine per kilogram of body weight. After 45 minutes, sports tests were conducted. No side effects were found in the test subjects during or after the competition (Gutiérrez-Hellín et al., 2016[30]). Another study using the same dosage showed an increase in the rate of fat oxidation, while the rate of carbohydrate oxidation during low-to-moderate intensity exercise was reduced. The increase in fat oxidation rate at the same exercise intensity and heart rate between the placebo group and the study group indicates an increased utilization of 7 grams of fat (Gutiérrez-Hellín and Del Coso, 2016[29]).

A study of fifteen professional cyclists showed that administration of 3 mg per kg of body weight of p-synephrine resulted in an increase in the rate of fat oxidation compared to placebo. However, it had no effect on energy expenditure or heart rate (Gutiérrez-Hellín et al., 2021[28]). Another study conducted by the same research team showed contradictory results. A test conducted on a group of 18 women who engaged in regular physical activity showed no change in the rate of fat oxidation. The dosage of p-synephrine remained the same. The only noticeable change was an increased eardrum temperature at rest. Heart rate also remained unchanged. The researchers suggested the gender of the subjects as a possible explanation for the difference in the results (Gutiérrez-Hellín et al., 2022[27]).

The effects of p-synephrine alone and in combination with bioflavonoids on self-esteem, blood pressure, heart rate and basal metabolism were studied on a group of 50 people. Administration of 50 mg of the pure compound or a mixture with naringin and hesperidin did not change resting heart rate, blood pressure or self-esteem. However, resting metabolism rate increased (Stohs et al., 2011[88]).

A non-obvious potential use for p-synephrine is as an ingredient in scalp rubs to reduce hair loss during hair styling. Many women experience hair loss during activities such as shampooing and brushing. Inside each hair follicle is an arrector pili muscle. These muscles are responsible for the piloerection commonly referred to as "goosebumps." Piloerection can be stimulated by mechanical, thermal or pharmacological stimuli. The pharmacological stimulus can be a response to the binding of catecholamines to the α1 adrenergic receptor. During piloerection, a much greater force must be applied to pull out the hair. The study involved applying a preparation containing synephrine, which is a selective α1 agonist, to the skin. The results showed that the application of a 10 % synephrine solution reduced the amount of hair loss during piloerection and increased the force needed to pull the hair out (McCoy et al., 2018[59]).

### Other studies

The described compound has a weak affinity for 5-hydroxytryptamine (5-HT) receptors. A study conducted on the rat aorta has shown that p-synephrine, by acting on α_1_, 5-HT_1D_ and 5-HT_2A_ receptors, induces aortic constriction. The compound was found to have no effect on the 5-HT_1B_ receptor (Hibino et al., 2009[35]). Another study confirmed the induction of thoracic rat aortic contraction by p-synephrine (Kim et al., 2019[44]).

p-Synephrine was tested on a mouse model of chronic social stress and on human microglia (HMC-3 cell line). It was shown that the tested compound reduced depressive behavior in mice. In microglia, it promoted a change in cell phenotype from pro-inflammatory (M1) to anti-inflammatory (M2). Affinity for the 5-HT_6_ receptor was proposed as the mechanism of action. Confirmation of p-synephrine's interaction with this receptor was obtained by Cellular Thermal Shift Assay (CETSA), molecular docking and coimmunoprecipitation. Binding of p-synephrine to the 5-HT_6_ receptor inhibited interaction with the FYN protein, reduced activation of the ERK1/2 pathway which reduced the neuroinflammatory response and had an anti-depressive effect on mice (Wang et al., 2025[98]).

In a mouse model, racemic p-synephrine was found to have antidepressant effects in a forced swimming and tail-hanging test. Preliminary results indicated an effect on the α_1_ receptors. Another study on enantiomers of p-synephrine showed that both compounds effectively inhibit norepinephrine reuptake and stimulate norepinephrine action in cerebral cortex. d-p-Synephrine showed stronger reuptake inhibition. In the tail suspension test, only d-p-synephrine proved effective. This contrasts the fact that l-p-synephrine interacts more strongly with the α_1_ receptors. It indicates that this enantiomer acts through a pathway other than interacting with the α_1_ receptors. It is noteworthy that administration of d-p-synephrine in doses higher than 10 mg/kg lost its effectiveness in the tail suspension test. It is also surprising that p-synephrine crossed the brain-blood barrier of mice. Repeated administration of d-p-synephrine did not induce the tolerance effect. Besides, administration of d-p-synephrine to mice at a dose of 300 mg/kg did not cause any acute toxic effects (Song et al., 1996[84]; Kim et al., 2001[45]). As early as 1965, it was reported that racemic p-synephrine inhibits norepinephrine reuptake (Burgen and Iversen, 1965[14]).

The effect of p-synephrine was examined on a dissected Wistar rat liver connected to a liver perfusion apparatus. The device enabled a controlled flow of a buffer through the organ. Administration of the tested compound resulted in higher hepatic lipase activity and increased excretion of free lipids by the liver. The study showed that p-synephrine increases lipolysis (Silva-Pereira et al., 2017[83]). Other studies on perfused rat livers have shown increases in glycolysis, glycogenolysis, and oxygen consumption. The proposed explanation for this effect is based on the impact of p-synephrine on adrenergic receptors (Peixoto et al., 2012[65]; De Oliveira et al., 2014[22]). A mouse study showed that feeding citrus p-synephrine to mice on a high-fat diet slowed adipose tissue proliferation and reduced the amount of mRNA for pro-inflammatory factors in perinephric adipose tissue. Surprisingly, administration of p-synephrine did not change the amount of serum lipids in a statistically significant way compared to the control group (Bai et al., 2024[5]).

In the literature, information on p-synephrine-drug interactions are scarce. An *in silico* study testing the possibility of an interaction between metformin and p-synephrine has been reported in the literature. Metformin is a drug used to treat type 2 diabetes, and a theoretical study showed that a non-covalent bond could be formed including the two compounds between an amino group and a hydroxyl group. It is possible that two conformers could be formed, one of which showed high bioavailability. The described study is an attempt to develop a more effective diabetes drug with a lower dosage (Prince Makarios Paul et al., 2024[70]). A different study in rats and rabbits looked at interactions with another diabetes drug, gliclazide. Healthy and diabetic animals were tested. A single administration of p-synephrine did not alter the pharmacokinetics and pharmacodynamics of gliclazide. Only repeated administration changed the pharmacodynamics of the drug - there was an increase in the reduction of glycemia in healthy and diseased rats and in healthy rabbits after administration of gliclazide. The observed change, according to the researchers, may be caused by the agonist effect of p-synephrine on β3 receptors (Vatsavai and Kilari, 2018[95]).

An unusual use of p-synephrine, is as a scavenger of toxic aldehydes such as acrolein and glyoxal. The study showed that the addition of p-synephrine to heat-treated foods allowed p-synephrine to bind acrolein. This effect also occurred *in vivo* in mouse studies for acrolein and glyoxal. This property suggests that the described substance may be a good food additive scavenging the mentioned two aldehydes (Jia et al., 2023[41]; Liang et al., 2024[51][52]; Zhong et al., 2024[110]). 

## Studies about Toxicity and Side Effects of p-Synephrine

There are many articles about the safety of synephrines as a group of substances. Table 2(Tab. 2) (References in Table 2: Arbo et al., 2009[3]; Bouchard et al., 2005[11]; Bui et al., 2006[13]; Cui et al., 2015[16]; Gutiérrez-Hellín and Del Coso 2016[29]; Gutiérrez-Hellín et al., 2016[30], 2021[28]; Hong et al., 2012[36]; Kaats et al., 2013[42]; Leão et al., 2021[50]; Min et al., 2005[61]; Nykamp et al., 2004[64]; Ribeiro et al., 2019[71], 2021[72]; Rossato et al., 2010[77]; Shara et al., 2018[82]; Stohs et al., 2011[88]) summarizes the selected toxicity studies and their results.

### Studies in experimental animals

Animal studies mainly focus on the activity of the described compound. The toxicity itself is often a side topic and poorly discussed.

In a study on male mice, animals were administered bitter orange extract by oral gavage. The animal groups studied had 9-10 animals each. They were given the extract at 400, 2000 and 4000 mg/kg and p-synephrine at 30 and 300 mg/kg. The p-synephrine content of the extract was confirmed by a previously validated HPLC/UV method. The study lasted for 28 days, during which no overt signs of toxicity, morbidity, or mortality were observed. The increase in body weight in p-synephrine-treated animals was compared to the other groups. Catalase activity increased, while the amount of malondialdehyde decreased and the amount of glutathione increased. Other markers of oxidative stress were normal (Arbo et al., 2009[3]).

The main limitations of this animal toxicity study are its duration, the testing of males only, and the possibility of different results in animals and humans. Side effects may appear after a longer period of time or due to interactions with other drugs. Teratogenicity and possible effects on future generations will also go unnoticed in such a short study.

### In vitro studies

Research on the cell line Caco-2 (immortalized human colorectal carcinoma) and freshly isolated cardiomyocytes from an adult rat showed that 3 hours of incubation in 1 mM or 500 µM solutions of p- and m-isomers did not result in visible cytotoxicity (Rossato et al., 2010[77]).

Research on human hepatocarcinoma cells (HepG2) showed that p-synephrine and caffeine alone do not reduce cell survival. Only simultaneous administration of both substances increased the percentage of dead cells. The combination of p-synephrine and caffeine increased the expression of genes related to apoptosis (BCL-2 and CASP9) and DNA repair (XPC), as well as decreased the expression of cell cycle control genes (CDKN1A). It was noted that the same relationship occurs for the amount of double-stranded DNA damage in cells (Leão et al., 2021[50]). Another study on HerG2 cells confirmed the lack of cytotoxicity even at higher concentrations of p-synephrine. It was also shown that 6 hours incubation of cells with the test substance increased the amount of ROS. Associated with this was a cellular response involving an increase in glutathione and activation of other mechanisms to eliminate ROS. p-Synephrine did not alter the expression of genes associated with DNA damage response (ATM, ATR, CHEK1, CHEK2, TP53, SIRT1), suggesting that cellular defense mechanisms were sufficient to prevent intracellular damage (Ribeiro et al., 2019[71]). 

It should be noted that studies on cell lines do not reflect the effects of a particular compound on the whole organism. A drug can also be toxic to only a selected organ.

### Studies in humans

Two human studies conducted by Gutiérrez-Hellín et al. showed no side effects after administration of p-synephrine at a dose of 3 mg per kg of body weight during sports activities (Gutiérrez-Hellín et al., 2021[28], 2022[27]). During the use of the supplement containing p-synephrine, the only possible side effect described by the authors was the possibility of pupil dilation after the liquid entered the eyes (McCoy et al., 2018[59]). Shara et al. study describing cardiovascular safety after oral administration of 49 mg of synephrine to 18 patients showed only a slight decrease in diastolic blood pressure (4.5 mmHg) 60 minutes after drug administration. Besides that, no other changes in heart rate, blood pressure, electrocardiograms or blood cell counts were noted (Shara et al., 2016[81]).

Bui et al., described the effects of a single administration of 900 mg of bitter orange extract standardized to 6 % synephrine. Blood pressure increased, with systolic peaking at 7,3 ± 4,6 mm Hg, diastolic at 2,6 ± 3,8 mm Hg, and a heart rate change of 4,2 ± 4,5 beats/min. However, the values were not large. The researchers noted that a limitation of their study was that it referred only to healthy subjects. In other groups of people such as the elderly, the sick or the obese, the results may be different (Bui et al., 2006[13]).

The study on 18 healthy volunteers showed no changes in corrected QT interval and blood pressure compared to placebo. The subjects were given 450 mg of bitter orange extract standardized to 27 mg of m- and p-synephrine. Measurements were taken at 1, 3, 5 and 8 hours after administration (Min et al., 2005[61]). Another study, conducted on 16 people for 15 days, consisted of administering 49 mg of p-synephrine daily and checking cardiovascular and hemodynamic parameters. No significant changes or adverse effects were registered (Shara et al., 2018[82]).

The longest human study associated with p-synephrine lasted 60 days. The 75 subjects (15 men and 60 women between 27 and 76 years) were divided into three groups: a placebo group, a group receiving 49 mg p-synephrine in bitter orange extract, and a group receiving an extract with naringin and hesperidin. The subjects took two servings daily. No side effects were observed in this study. None of the participants reported adverse reactions. A limitation of the study was that patients were examined only before and after the end of taking p-synephrine. No examinations were conducted during the study, therefore, it is not known what was happening to the subjects while actively taking p-synephrine. It is also not known whether the subjects took their medications appropriately, as plasma p-synephrine levels were not measured during the study (Kaats et al., 2013[42]).

The study on 10 healthy adults (including three women) aged 20 to 31 years examined how oral administration of 21 mg of p-synephrine and 304 mg of caffeine would affect the human body during moderately intense exercise on a bicycle ergometer (for 30 minutes). The concentration of the tested compounds in the dietary supplement and the subjects' plasma was confirmed by LC/MS-MS. Plasma concentrations of p-synephrine and caffeine were measured for 12 hours, and vital signs, serum electrolytes, oxygen consumption and subjective sensation of exertion during exercise were monitored. No significant adverse events were reported. Diastolic blood pressure after exercise was higher after the supplement (mean 71.7 mmHg) than after placebo (63.0 mmHg). There were no significant differences in heart rate, systolic blood pressure or body temperature. Postprandial plasma glucose levels rose to 121.0 mg/dl after supplement administration and exercising compared to 103.7 mg/dl after placebo and exercising. An examination of subjective feelings showed that study participants rated exercise as less tiring after taking the supplement compared to the placebo group (Haller et al., 2008[31]).

All human studies have been carried out on healthy and adult subjects. This makes it impossible to predict how p-synephrine will work on people who are sick, or belong to other age groups. Human studies have been conducted on groups with a female-to-male ratio other than 1:1. The effect of p-synephrine may vary by gender, this property has not been sufficiently studied.

### Case reports (humans)

A meta-analysis summarizing 18 articles involving a total of 341 study subjects included oral administration of p-synephrine at doses ranging from 6 to 214 mg. Both systolic and diastolic blood pressure increased significantly after long-term p-synephrine use (6.37 mmHg for systolic pressure and 4.33 mmHg for diastolic pressure). Weight loss in the synephrine group was insignificant after long-term treatment and had no effect on body composition parameters. Based on the review, it was determined that the use of the substance described may elevate blood pressure and heart rate, which may increase the risk of stroke and heart attack in predisposed individuals (Koncz et al., 2022[48]).

A review summarizing 35 people who experienced side effects that may be related to p-synephrine use indicates that the most common symptoms appeared on the cardiovascular side. Many patients took several supplements at once, making it impossible to pinpoint a single cause of symptoms. The review suggests that p-synephrine use may be associated with a higher risk of sudden cardiovascular-related illnesses such as heart attacks and arrhythmias. Limitations of the review include the small number of cases and uncertainty about the quality and purity of the dietary supplements that patients took (de Jonge et al., 2023[21]).

Three selected cases of myocardial infractions covered in the above review are discussed below. They all describe similar cases of people who took several substances at the same time.

One case is a 38-year-old man who suffered an ischemic stroke. The incident was preceded by numerous central nervous system symptoms such as double vision and dizziness. The patient was not in a stroke risk group. For a week before the incident, the patient had been taking a dietary supplement for weight loss. One tablet contained 6 mg of synephrine and 200 mg of caffeine alkaloids. The patient had been taking 1-2 tablets daily. At the end of the week-long hospitalization, the patient had almost completely recovered (Bouchard et al., 2005[11]).

In 2002, a case of acute lateral-wall myocardial infarction in a 55-year-old woman was described. The incident coincided with the use of a dietary supplement containing bitter orange. The patient's myocardial infarction began with standard clinical symptoms. The patient was hospitalized and received thrombolytic treatment. For the past year, she has been taking a dietary supplement for weight loss. The formula contained 300 mg of bitter orange and other additives such as green tea and guarana. The mentioned additives were a source of caffeine. The patient had no previous illnesses, and she had a history of smoking (Nykamp et al., 2004[64]).

A 22-year-old man with no medical history was diagnosed with a ST-segment elevation myocardial infarction (STEMI). The symptoms of the infarction occurred during a basketball game, 2 hours after taking a dietary supplement. The patient had been taking two supplements over the past year, which included synephrine and bitter orange extract. Researchers linked the STEMI to the patient's intake of the supplements (Unnikrishnan et al., 2018[94]).

Cases worthy of note are two patients admitted to the same emergency department within 48 hours. The first patient was a 28-year-old man with a history of HIV, mental illness and drug addiction. He was found unconscious on the street in a hyperthermic state. Among other things, cocaine, its metabolites and synephrine were detected in his serum. The synephrine concentration was 3010 ng/mL. The man was transferred to a rehabilitation center nearly three months after admission to the emergency department due to multiple complications. The second case involved a 24-year-old woman with a history of drug use. She was hospitalized after using crack and heroin. She had sinus tachycardia and developed hyperthermia while in the unit. Among other things, fentanyl and synephrine were detected in her serum. The synephrine concentration was 37700 ng/mL. Three days after the report, the patient left the hospital on her own request, with no neurological deficit. The cases described are noteworthy because of the huge concentrations of synephrine detected in patients. Most likely, synephrine was used as an adulterant in street drugs. Hyperthermia that proved difficult to control was most likely caused by synephrine. The amount of the substance in the plasma exceeded many times the amount consumed by people taking dietary supplements (Choe et al., 2023[15]).

The main drawback of the case studies is the lack of information as to the exact purity of p-synephrine confirmed by an independent analytical lab. Information on the doses taken is also unclear. Often patients themselves do not know exactly what doses they have taken, taking dietary supplements irregularly. The unknown duration of exposure and additives make it very difficult to link p-synephrine to an increased risk of a particular disease.

## Conclusions

p-Synephrine is characterized by a broad spectrum of activity. It is advertised as a weight loss agent, which is supported by few scientific research papers. Studies on cell lines, animals, and humans do not indicate its toxicity at low doses. Therefore, short-term use of this compound in low doses is safe for healthy people. Adverse effects of p-synephrine have been reported in the literature; however, they relate to the use of large doses or the combination of this compound with other stimulants (such as caffeine). The described harmful effects may be due to diseases unrelated to p-synephrine use. Research on its long-term effects on human health is lacking. Because it is found in citrus fruit products, it is part of the diet for those who consume these fruits. It also plays a role in neuropharmacology, however, it has not been sufficiently studied. Due to the isomeric substitution of the hydroxyl group to the aromatic ring, there may be confusion in the literature. The presence of a chiral carbon atom means that inaccuracies regarding the single enantiomer or racemate used are present in the studies. Available studies indicate that plant-derived p-synephrine is safe for healthy people in low doses with short-term use. Further studies are needed to thoroughly investigate the effects of this compound and its isomers.

## Declaration

### Methodology of the review

The manuscript was based on a conducted review of online databases of scientific articles such as PubMed, Embase, Scopus, Web of Science, and Google Scholar. The key search term used was '*p-synephrine*'. The relevance of the articles was determined by reviewing their abstracts. The focus was mainly on articles related to* in vitro* and *in vivo *studies. The manuscript includes articles published before 2025.

### Authors' contributions

Conceptualization, MK, KB and AK; writing-original draft preparation, MK, KB and AK; writing-review and editing, MK, KB and AK; supervision, AK All authors have read and agreed to the published version of the manuscript.

### Competing interests

The authors declare that they have no conflict of interest.

### Acknowledgment

The graphical abstract was drawn using images from Servier Medical Art (https://smart.servier.com/citation-sharing/). Servier Medical Art by Servier is licensed under a Creative Commons Attribution 4.0 public License (CC BY) (https://creativecommons.org/licenses/by/4.0/).

## Figures and Tables

**Table 1:**
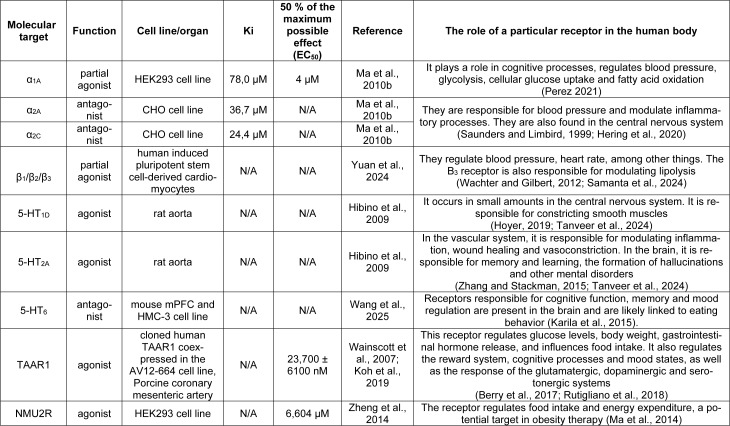
Table 1: The effect of p-synephrine on individual receptors

**Table 2:**
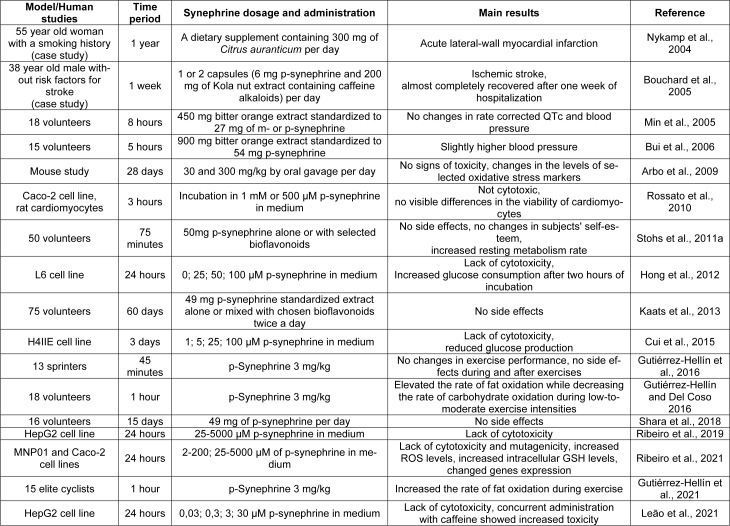
Summary of the effect of p-synephrine in selected scientific papers

**Figure 1:**
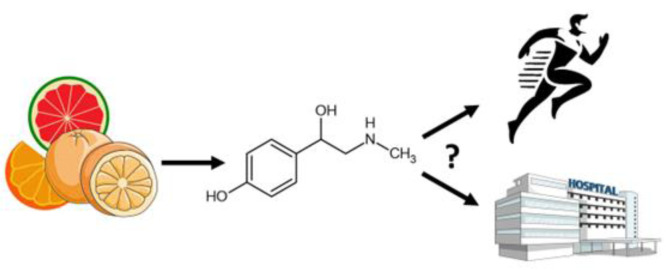
Graphical abstract

## References

[1] Allison DB, Cutter G, Poehlman ET, Moore DR, Barnes S (2005). Exactly which synephrine alkaloids does Citrus aurantium (bitter orange) contain?. Int J Obes.

[2] Arbo MD, Larentis ER, Linck VM, Aboy AL, Pimentel AL, Henriques AT (2008). Concentrations of p-synephrine in fruits and leaves of Citrus species (Rutaceae) and the acute toxicity testing of Citrus aurantium extract and p-synephrine. Food Chem Toxicol.

[3] Arbo MD, Schmitt GC, Limberger MF, Charão MF, Moro ÂM, Ribeiro GL (2009). Subchronic toxicity of Citrus aurantium L. (Rutaceae) extract and p-synephrine in mice. Regul Toxicol Pharmacol.

[4] Bader M, Lang T, Lang R, Hofmann T (2017). Synephrine as a specific marker for orange consumption. J Agric Food Chem.

[5] Bai J, Tan X, Tang S, Liu X, Shao L, Wang C (2024). Citrus p-synephrine improves energy homeostasis by regulating amino acid metabolism in HFD-induced Mice. Nutr.

[6] Bartley GE, Breksa AP, Ishida BK (2010). PCR amplification and cloning of tyrosine decarboxylase involved in synephrine biosynthesis in Citrus. N Biotechnol.

[7] Bergmann ED, Sulzbacher M (1951). A new synthesis of 1-(m- and p-hydroxyphenyl)-2-methylaminoethanol (m- and p-sympathol). J Org Chem.

[8] Berry MD, Gainetdinov RR, Hoener MC, Shahid M (2017). Pharmacology of human trace amine-associated receptors: Therapeutic opportunities and challenges. Pharmacol Ther.

[9] Bhattacharyya S, Luan J, Farooqi IS, Keogh J, Montague C, Brennand J (2004). Studies of the neuromedin U-2 receptor gene in human obesity: evidence for the existence of two ancestral forms of the receptor. J Endocrinol.

[10] Bouchard N, Hoffman RS, Haigney M (2004). Synephrine is not neo-synephrine [5] (multiple letters). Mayo Clin Proc.

[11] Bouchard NC, Howland MA, Greller HA, Hoffman RS, Nelson LS (2005). Ischemic stroke associated with use of an ephedra-free dietary supplement containing synephrine. Mayo Clin Proc.

[12] Brewer K, Machin JJ, Maylin G, Fenger C, Morales-Briceño A, Neidhart MM (2022). Case report: Synephrine, a plant substance yielding classic environmental clusters of hay related identifications in equine urine. Drug Test Anal.

[13] Bui LT, Nguyen DTT, Ambrose PJ (2006). Blood pressure and heart rate effects following a single dose of bitter orange. Ann Pharmacother.

[14] Burgen ASV, Iversen LL (1965). The inhibition of noradrenaline uptake by sympathomimetic amines in the rat isolated heart. Br J Pharmacol Chemother.

[15] Choe AJ, Ellison R, Ramaswamy SR, Schult RF, Gerona R, Nacca N (2023). Profound hyperthermia associated with fentanyl and cocaine use with suspected synephrine adulteration. J Emerg Med.

[16] Cui Z, Lee Y, Lee Y, Park D (2015). p-Synephrine suppresses glucose production but not lipid accumulation in H4IIE liver cells. J Med Food.

[17] D’Andrea G, Granella F, Leone M, Perini F, Farruggio A, Bussone G (2006). Abnormal platelet trace amine profiles in migraine with and without aura. Cephalalgia.

[18] D’Andrea G, Terrazzino S, Fortin D, Cocco P, Balbi T, Leon A (2003). Elusive amines and primary headaches: Historical background and prospectives. Neurol Sci.

[19] D’Andrea G, Terrazzino S, Fortin D, Farruggio A, Rinaldi L, Leon A (2003). HPLC electrochemical detection of trace amines in human plasma and platelets and expression of mRNA transcripts of trace amine receptors in circulating leukocytes. Neurosci Lett.

[20] Daina A, Michielin O, Zoete V (2017). SwissADME: a free web tool to evaluate pharmacokinetics, drug-likeness and medicinal chemistry friendliness of small molecules. Sci Rep.

[21] de Jonge MLL, Kieviet LC, Sierts M, Egberink LB, van der Heyden MAG (2023). Review of case reports on adverse events related to pre-workout supplements containing synephrine. Cardiovasc Toxicol.

[22] de Oliveira AL, Comar JF, De Sá-Nakanishi AB, Peralta RM, Bracht A (2014). The action of p-synephrine on hepatic carbohydrate metabolism and respiration occurs via both Ca2+-mobilization and cAMP production. Mol Cell Biochem.

[23] Dong X (2018). Current strategies for brain drug delivery. theranostics. Theranostics.

[24] Dragull K, Breksa AP, Cain B (2008). Synephrine content of juice from Satsuma mandarins (Citrus unshiu Marcovitch). J Agric Food Chem.

[25] Fugh-Berman A, Myers A (2004). Citrus aurantium, an ingredient of dietary supplements marketed for weight loss: current status of clinical and basic research. Exp Biol Med (Maywood).

[26] Guo LX, Chen G, Yin ZY, Zhang YH, Zheng XX (2019). p-Synephrine exhibits anti-adipogenic activity by activating the Akt/GSK3β signaling pathway in 3T3-L1 adipocytes. J Food Biochem.

[27] Gutiérrez-Hellín J, Aguilar-Navarro M, Ruiz-Moreno C, Muñoz A, Amaro-Gahete FJ, Posada-Ayala M (2022). Effect of p-synephrine on fat oxidation rate during exercise of increasing intensity in healthy active women. Nutrition.

[28] Gutiérrez-Hellín J, Baltazar-Martins G, Rodríguez I, Lara B, Ruiz-Moreno C, Aguilar-Navarro M (2021). p-Synephrine, the main protoalkaloid of Citrus aurantium, raises fat oxidation during exercise in elite cyclists. Eur J Sport Sci.

[29] Gutiérrez-Hellín J, Del Coso J (2016). Acute p-synephrine ingestion increases fat oxidation rate during exercise. Br J Clin Pharmacol.

[30] Gutiérrez-Hellín J, Salinero JJ, Abían-Vicen J, Areces F, Lara B, Gallo C (2016). Acute consumption of p-synephrine does not enhance performance in sprint athletes. Appl Physiol Nutr Metab.

[31] Haller CA, Duan M, Jacob P, Benowitz N (2008). Human pharmacology of a performance-enhancing dietary supplement under resting and exercise conditions. Br J Clin Pharmacol.

[32] Hengstmann JH, Aulepp H (1978). Pharmacokinetics and metabolism of 3H-synephrine. Arzneimittelforschung.

[33] Henning N, Kellermann TA, Smith C (2024). Effect of chronic dolutegravir administration on the trace amine profile in wistar rats. Drugs R D.

[34] Hering L, Rahman M, Potthoff SA, Rump LC, Stegbauer J (2020). Role of α2-Adrenoceptors in hypertension: focus on renal sympathetic neurotransmitter release, inflammation, and sodium homeostasis. Front Physiol.

[35] Hibino T, Yuzurihara M, Kase Y, Takeda A (2009). Synephrine, a component of Evodiae Fructus, constricts isolated rat aorta via adrenergic and serotonergic receptors. J Pharmacol Sci.

[36] Hong NY, Cui ZG, Kang HK, Lee DH, Lee YK, Park DB (2012). p-Synephrine stimulates glucose consumption via AMPK in L6 skeletal muscle cells. Biochem Biophys Res Commun.

[37] Hou X, Yan D, Wu Z, Mao L, Wang H, Guo Y (2024). Discovery of dolutegravir derivative against liver cancer via inducing autophagy and DNA damage. Molecules.

[38] Hoyer D, Tricklebank MD, Daly E (2019). Serotonin receptors nomenclature. The serotonin system. History, neuropharmacolology, and pathology.

[39] Ianno V, Clevers S, Négrier P, Dupray V, Coquerel G, Espeau P (2020). p-Synephrine enantiomers: binary phase diagram, crystal structure and kinetic stability of a metastable conglomerate monitored by nonlinear optics. CrystEngComm.

[40] Ishida M, Takekuni C, Nishi K, Sugahara T (2022). p-Synephrine suppresses inflammatory responses in lipopolysaccharide-stimulated RAW264.7 cells and alleviates systemic inflammatory response syndrome in mice. Food Funct.

[41] Jia M, Gu H, Lu Y, Lv L (2023). Effects of hesperidin combined with synephrine on the capture of acrolein in a mouse model, or in humans by citrus consumption. Food Funct.

[42] Kaats GR, Miller H, Preuss HG, Stohs SJ (2013). A 60 day double-blind, placebo-controlled safety study involving Citrus aurantium (bitter orange) extract. Food Chem Toxicol.

[43] Karila D, Freret T, Bouet V, Boulouard M, Dallemagne P, Rochais C (2015). Therapeutic potential of 5-HT6 receptor agonists. J Med Chem.

[44] Kim JJ, Kim K, Jung YR, Bian Y, Ngo T, Bae ON (2019). Co-existence of hypertensive and anti-hypertensive constituents, synephrine, and nobiletin in Citrus unshiu Peel. Molecules.

[45] Kim KW, Kim HD, Jung JS, Woo RS, Kim HS, Suh HW (2001). Characterization of antidepressant-like effects of p-synephrine stereoisomers. Naunyn Schmiedebergs Arch Pharmacol.

[46] Koh AHW, Chess-Williams R, Lohning AE (2019). Differential mechanisms of action of the trace amines octopamine, synephrine and tyramine on the porcine coronary and mesenteric artery. Sci Rep.

[47] Koh AHW, Chess-Williams R, Lohning AE (2021). Racemic synephrine found in Citrus aurantium-listing pre-workout supplements suggests a non-plant-based origin. Drug Test Anal.

[48] Koncz D, Tóth B, Bahar MA, Roza O, Csupor D (2022). The safety and efficacy of citrus aurantium (bitter orange) extracts and p-synephrine: a systematic review and meta-analysis. Nutrients.

[49] Kraboth Z, Kalman B (2023). ß-Adrenoreceptors in human cancers. Int J Mol Sci.

[50] Leão TK, Ribeiro DL, Machado ART, Costa TR, Sampaio SV, Antunes LMG (2021). Synephrine and caffeine combination promotes cytotoxicity, DNA damage and transcriptional modulation of apoptosis-related genes in human HepG2 cells. Mutat Res Toxicol Environ Mutagen.

[51] Liang Y, Du R, Zhao X, Xu Y, Xiang Q, Wu H (2024). Scavenging glyoxal and methylglyoxal by synephrine alone or in combination with neohesperidin at high temperatures. J Agric Food Chem.

[52] Liang Y, Zhao X, Xu Y, Lu Y, Lv L (2024). Scavenging glyoxal and methylglyoxal by synephrine and neohesperidin from flowers of Citrus aurantium L. var. amara Engl. in mice and humans. J Agric Food Chem.

[53] Lin LY, Peng CC, Huang YP, Chen KC, Peng RY (2023). p-Synephrine indicates internal maturity of Citrus grandis (L.) Osbeck cv. Mato Peiyu-reclaiming functional constituents from nonedible parts. Molecules.

[54] Lindemann L, Hoener MC (2005). A renaissance in trace amines inspired by a novel GPCR family. Trends Pharmacol Sci.

[55] Ma G, Bavadekar SA, Schaneberg BT, Khan IA, Feller DR (2010). Effects of synephrine and beta-phenethylamine on human alpha-adrenoceptor subtypes. Planta Med.

[56] Ma G, Bavadekar SA, Schaneberg BT, Khan IA, Feller DR (2010). Effects of Synephrine and β-phenethylamine on human α-adrenoceptor subtypes. Planta Med.

[57] Ma ML, Li M, Gou JJ, Ruan TY, Jin HS, Zhang LH (2014). Design, synthesis and biological activity of flavonoid derivatives as selective agonists for neuromedin U 2 receptor. Bioorg Med Chem.

[58] Malesuik MD, Pereira CS, Kaefer CL, Bordim JMT, Paula FR, Paim CS (2024). Synephrine photodegradation study: Degradation kinetic, in silico and LC-ESI-MS analysis of major degradation product, and in vitro toxicological study. J Photochem Photobiol A Chem.

[59] McCoy J, Goren A, Kovacevic M, Situm M, Stanimirovic A, Shapiro J (2018). Styling without shedding: Novel topical formula reduces hair shedding by contracting the arrector pili muscle. Dermatol Ther.

[60] Midgley JM, Thonoor CM, Drake AF, Williams CM, Koziol AE, Palenik GJ (1989). The resolution and absolute configuration by X-ray crystallography of the isomeric octopamines and synephrines. J Chem Soc Perkin Trans 2.

[61] Min B, Cios D, Kluger J, White CM (2005). Absence of QTc-interval-prolonging or hemodynamic effects of a single dose of bitter-orange extract in healthy subjects. Pharmacotherapy.

[62] Nair PC, Chalker JM, Mckinnon RA, Langmead CJ, Gregory KJ, Bastiampillai T (2022). Trace Amine-Associated Receptor 1 (TAAR1): molecular and clinical insights for the treatment of schizophrenia and related comorbidities. ACS Pharmacol Transl Sci.

[63] Nasir JM, Durning SJ, Ferguson M, Barold HS, Haigney MC (2004). Exercise-induced syncope associated with QT prolongation and ephedra-free xenadrine. Mayo Clin Proc.

[64] Nykamp DL, Fackih MN, Compton AL (2004). Possible association of acute lateral-wall myocardial infarction and bitter orange supplement. Ann Pharmacother.

[65] Peixoto JS, Comar JF, Moreira CT, Soares AA, De Oliveira AL, Bracht A (2012). Effects of Citrus aurantium (bitter orange) fruit extracts and p-synephrine on metabolic fluxes in the rat liver. Molecules.

[66] Pellati F, Benvenuti S (2007). Chromatographic and electrophoretic methods for the analysis of phenetylamine alkaloids in Citrus aurantium. J Chromatogr A.

[67] Pellati F, Cannazza G, Benvenuti S (2010). Study on the racemization of synephrine by off-column chiral high-performance liquid chromatography. J Chromatogr A.

[68] Perez DM (2021). Current Developments on the role of α1-adrenergic receptors in cognition, cardioprotection, and metabolism. Front Cell Dev Biol.

[69] Piattoly TJ (2022). Dietary supplement safety: risk vs reward for athletes. Oper Tech Sports Med.

[70] Prince Makarios Paul S, Parimala Devi D, Nancy Sukumar A, Praveena G, Jeba Beula R, Abiram A (2024). Theoretical insights on the interaction between p-synephrine and Metformin: A DFT, QTAIM and Drug-Likeness investigation. Comput Theor Chem.

[71] Ribeiro DL, Machado ART, da Silva Machado C, Santos PW da S, Aissa AF, Barcelos GRM (2019). Analysis of the cytotoxic, genotoxic, mutagenic, and pro-oxidant effect of synephrine, a component of thermogenic supplements, in human hepatic cells in vitro. Toxicology.

[72] Ribeiro DL, Machado ART, Machado C, Ferro Aissa A, Dos Santos PW, Barcelos GRM (2021). p-Synephrine induces transcriptional changes via the cAMP/ PKA pathway but not cytotoxicity or mutagenicity in human gastrointestinal cells. J Toxicol Environ Heal Part A.

[73] Roh KB, Kim IH, Kim YS, Lee M, Lee JA, Jung E (2014). Synephrine inhibits eotaxin-1 expression via the STAT6 signaling pathway. Molecules.

[74] Rosa F, Négrier P, Corvis Y, Espeau P (2016). Crystal structure determination and thermal behavior upon melting of p-synephrine. Thermochim Acta.

[75] Rossato LG, Costa VM, De Pinho PG, Carvalho F, De Lourdes Bastos M, Remião F (2011). Structural isomerization of synephrine influences its uptake and ensuing glutathione depletion in rat-isolated cardiomyocytes. Arch Toxicol.

[76] Rossato LG, Costa VM, Limberger RP, Bastos M de L, Remião F (2011). Synephrine: From trace concentrations to massive consumption in weight-loss. Food Chem Toxicol.

[77] Rossato LG, de Pinho PG, Silva R, Carmo H, Carvalho F, Bastos M de L (2010). Development and validation of a GC/IT-MS method for simultaneous quantitation of para and meta-synephrine in biological samples. J Pharm Biomed Anal.

[78] Rutigliano G, Accorroni A, Zucchi R (2018). The case for TAAR1 as a modulator of central nervous system function. Front Pharmacol.

[79] Samanta S, Bagchi D, Bagchi M (2024). Physiological and metabolic functions of the β3-adrenergic receptor and an approach to therapeutic achievements. J Physiol Biochem.

[80] Saunders C, Limbird LE (1999). Localization and trafficking of α2-adrenergic receptor subtypes in cells and tissues. Pharmacol Ther.

[81] Shara M, Stohs SJ, Mukattash TL (2016). Cardiovascular safety of oral p-synephrine (bitter orange) in healthy subjects: a randomized placebo-controlled cross-over clinical trial. Phytother Res.

[82] Shara M, Stohs SJ, Smadi MM (2018). Safety evaluation of p-synephrine following 15 days of oral administration to healthy subjects: A clinical study. Phytother Res.

[83] Silva-Pereira JF da, Valoto AL de O, Bracht L, Gonçalves G de A, Peralta RM, Bracht A (2017). The action of p-synephrine on lipid metabolism in the perfused rat liver. J Biosci Med.

[84] Song DK, Suh HW, Jung JS, Wie MB, Son KH, Kim YH (1996). Antidepressant-like effects of p-synephrine in mouse models of immobility tests. Neurosci Lett.

[85] Stewart I, Newhall WF, Edwards GJ (1964). The isolation and identification of l-synephrine in the leaves and fruit of citrus. J Biol Chem.

[86] Stohs SJ (2013). Problems with Citrus aurantium information in “A Review on Botanical Species and Chemical Compounds with Appetite Suppressing Properties for Body Weight Control.”. Plant Foods Hum Nutr.

[87] Stohs SJ, Preuss HG (2012). Stereochemical and pharmacological differences between naturally occurring p-synephrine and synthetic p-synephrine. J Funct Foods.

[88] Stohs SJ, Preuss HG, Keith SC, Keith PL, Miller H, Kaats GR (2011). Effects of p-synephrine alone and in combination with selected bioflavonoids on resting metabolism, blood pressure, heart rate and self-reported mood changes. Int J Med Sci.

[89] Stohs SJ, Preuss HG, Shara M (2011). A review of the receptor-binding properties of p-synephrine as related to its pharmacological effects. Oxid Med Cell Longev.

[90] Suzuki O, Matsumoto T, Oya M, Katsumata Y (1979). Oxidation of synephrine by type A and type B monoamine oxidase. Experientia.

[91] Taheri R, Hamzkanlu N, Rezvani Y, Niroumand S, Samandar F, Amiri-Tehranizadeh Z (2022). Exploring the HSA/DNA/lung cancer cells binding behavior of p-Synephrine, a naturally occurring phenyl ethanol amine with anti-adipogenic activity: multi spectroscopic, molecular dynamic and cellular approaches. J Mol Liq.

[92] Tanveer J, Baig A, Rubeen R, Qureshi SR, Bashir N, Khan K, Fatima-Shad K (2024). Unlocking the mysteries: serotonin receptor networks explored. Serotonin - neurotransmitter and hormone of brain, bowels and blood.

[93] Tette PAS, Guidi LR, Bastos EMAF, Fernandes C, Gloria MBA (2017). Synephrine - a potential biomarker for orange honey authenticity. Food Chem.

[94] Unnikrishnan D, Annam R, Jacob A, Thyagarajan B, Farrugia P (2018). STEMI in a young male after use of synephrine-containing dietary supplement. Case Reports Cardiol.

[95] Vatsavai LK, Kilari EK (2018). Interaction of p-synephrine on the pharmacodynamics and pharmacokinetics of gliclazide in animal models. J Ayurveda Integr Med.

[96] Wachter SB, Gilbert EM (2012). Beta-adrenergic receptors, from their discovery and characterization through their manipulation to beneficial clinical application. Cardiology.

[97] Wainscott DB, Little SP, Yin T, Tu Y, Rocco VP, He JX (2007). Pharmacologic characterization of the cloned human trace amine-associated receptor1 (TAAR1) and evidence for species differences with the rat TAAR1. J Pharmacol Exp Ther.

[98] Wang S, Li Q, Deng D, Xie Z, Cao K, Zhang F (2025). Facilitating microglia M2 polarization alleviates p-Synephrine-induced depressive-like behaviours in CSDS mice via the 5-HT6R-FYN-ERK1/2 pathway. Int Immunopharmacol.

[99] Wang YL, Lin SX, Wang Y, Liang T, Jiang T, Liu P (2023). p-Synephrine ameliorates alloxan-induced diabetes mellitus through inhibiting oxidative stress and inflammation via suppressing the NF-kappa B and MAPK pathways. Food Funct.

[100] Watson DG, Midgley JM, Chen RN, Huang W, Bain GM, McDonald NM (1990). Analysis of biogenic amines and their metabolites in biological tissues and fluids by gas chromatography—negative ion chemical ionization mass spectrometry (GC-NICIMS). J Pharm Biomed Anal.

[101] Wheaton TA, Stewart I (1969). Biosynthesis of synephrine in citrus. Phytochemistry.

[102] Wu Q, Li R, Soromou LW, Chen N, Yuan X, Sun G (2014). p-Synephrine suppresses lipopolysaccharide-induced acute lung injury by inhibition of the NF-κB signaling pathway. Inflamm Res.

[103] Wu Y, Zeng L, Zhao S (2021). Ligands of adrenergic receptors: A structural point of view. Biomolecules.

[104] Xanthopoulos A, Daskalopoulou I, Frountzi S, Papadimitriou E (2021). A systematic review on the role of adrenergic receptors in angiogenesis regulation in health and disease. Int J Transl Med.

[105] Xu WW, Zheng CC, Huang YN, Chen WY, Yang QS, Ren JY (2018). Synephrine hydrochloride suppresses esophageal cancer tumor growth and metastatic potential through inhibition of galectin-3-AKT/ERK signaling. J Agric Food Chem.

[106] Yuan X, Yu T, Zhang Z, Li S (2024). Non-invasive assessment of proarrhythmic risks associated with isoprenaline and the dietary supplement ingredient synephrine using human induced pluripotent stem cell-derived cardiomyocytes. Front Cardiovasc Med.

[107] Zhang G, Stackman RW (2015). The role of serotonin 5-HT2A receptors in memory and cognition. Front Pharmacol.

[108] Zheng X, Guo L, Wang D, Deng X (2014). p-Synephrine: a novel agonist for neuromedin U2 receptor. Biol Pharm Bull.

[109] Zhong C, Yang X, Niu J, Zhou X, Zhou J, Pan G (2024). Transcriptome analysis of Citrus aurantium L. to study synephrine biosynthesis during developmental stages. PeerJ.

[110] Zhong Y, Liang Y, Jia M, Si B, Lv L (2024). Synephrine, as a scavenger and promoter, cooperates with hesperidin to reduce acrolein levels. Food Chem.

